# Artificial Intelligence-Based Human–Computer Interaction Technology Applied in Consumer Behavior Analysis and Experiential Education

**DOI:** 10.3389/fpsyg.2022.784311

**Published:** 2022-04-06

**Authors:** Yanmin Li, Ziqi Zhong, Fengrui Zhang, Xinjie Zhao

**Affiliations:** ^1^Pan Tianshou College of Architecture, Art and Design, Ningbo University, Ningbo, China; ^2^Department of Management, The London School of Economics and Political Science, London, United Kingdom; ^3^College of Life Science, Sichuan Agricultural University, Yaan, China; ^4^School of Software and Microelectronics, Peking University, Beijing, China

**Keywords:** image recognition, human-computer interaction, customer psychology, behavior analysis, deep neutral network

## Abstract

In the course of consumer behavior, it is necessary to study the relationship between the characteristics of psychological activities and the laws of behavior when consumers acquire and use products or services. With the development of the Internet and mobile terminals, electronic commerce (E-commerce) has become an important form of consumption for people. In order to conduct experiential education in E-commerce combined with consumer behavior, courses to understand consumer satisfaction. From the perspective of E-commerce companies, this study proposes to use artificial intelligence (AI) image recognition technology to recognize and analyze consumer facial expressions. First, it analyzes the way of human–computer interaction (HCI) in the context of E-commerce and obtains consumer satisfaction with the product through HCI technology. Then, a deep neural network (DNN) is used to predict the psychological behavior and consumer psychology of consumers to realize personalized product recommendations. In the course education of consumer behavior, it helps to understand consumer satisfaction and make a reasonable design. The experimental results show that consumers are highly satisfied with the products recommended by the system, and the degree of sanctification reaches 93.2%. It is found that the DNN model can learn consumer behavior rules during evaluation, and its prediction effect is increased by 10% compared with the traditional model, which confirms the effectiveness of the recommendation system under the DNN model. This study provides a reference for consumer psychological behavior analysis based on HCI in the context of AI, which is of great significance to help understand consumer satisfaction in consumer behavior education in the context of E-commerce.

## Introduction

Consumer behavior is a science that studies the characteristics of mental activities and behavioral laws that consumers take place in the process of acquiring, using, consuming, and disposing of products and services. From the perspective of marketing, this subject provides an understanding of consumer behavior because “marketing is a subject that tries to influence consumer behavior.” However, with the continuous development of the Internet and mobile terminals, electronic commerce (E-commerce) has penetrated into all the aspects of the lives of people, and online shopping has become the main form of the consumption of people (Qian et al., 2018). According to a statistical report released by the China Internet Network Information Center, the number of online shopping users in China has reached 710 million as of March 2020, with an increase of 16.4% compared with that at the end of 2018 and accounting for 78.6% of the overall Internet users (Campbell and Winterich, 2018; Lamberton, 2019). In 2019, online retail sales of China reached 10.63 trillion yuan, of which physical goods were 8.52 trillion yuan, accounting for 20.7% of the total retail sales of consumer goods. From January to February 2020, the online retail sales of physical goods in China increased by 3.0% year on year, achieving growth against the trend, accounting for 21.5% of the total retail sales of consumer goods. In addition, it was an increase of 5.0% over the same period last year. People can obtain great convenience through the Internet, especially the emergence of shopping mobile terminals, so that many consumers can browse goods at any time, get what they need, and bring great convenience to the lives of people. However, this has caused difficulties in understanding the changes in consumer psychological activities in the course. Therefore, it is proposed to use artificial intelligence (AI) technology to understand the changes in consumer psychological behavior in the E-commerce environment (Cichy and Kaiser, 2019).

The development of AI has also brought more opportunities to many fields. Especially after the cooperation between AI and business, whether in finance or the Internet industry, it has brought more possibilities for the development of enterprises (Kell et al., 2018). Some experts pointed out that with the widespread application of AI in 2018, the consumer experience has been greatly improved. AI not only realizes daily interaction, but also allows people to enjoy immersive experience technology through related devices (Kim and Cho, 2019). The AI system is adopted by the recommendation engine of Amazon to help and improve the original recommendation services, so that consumers can choose very attractive-related recommendation products. Currently, this technology is also considered a very reliable shopping consultant. The continuous development of human–computer interaction (HCI) and deep learning also brings more possibilities for data acquisition and analysis and provides more technical support for marketing and meeting the needs of more people (Feng et al., 2019). Therefore, students can be taught experientially in the course of consumer behavior with AI technology to help them understand the changes in the minds of users in a timely manner.

In summary, there are reliable internal laws in the buying behavior of consumers and their consumption levels and preferences will be affected by psychology. In the education courses of consumer behavior, mastering consumer behavior and psychology can better formulate marketing strategies and provide consumers with precise services. From the perspective of E-commerce, AI is used to identify and analyze the facial expressions of consumers and to obtain consumer satisfaction with products through HCI, so as to realize the experiential education of students in the consumer behavior course. Finally, the improved deep neural network (DNN) is used to predict the psychological behaviors of consumers, achieve precision marketing, and improve operational efficiency, which is of key significance to promote the understanding of students to consumer experience in the course.

The innovative point of the work is to effectively judge the consumer psychology of consumers through the behavior of HCI and then promote the educational experience of students in the course of consumer behavior. The work is developed in four parts. The first part introduces the application of HCI technology in consumer behavior education under the background of E-commerce. The second part is to analyze the application of neural network technology and establish an experimental model. The third part is to introduce the experimental settings and explain the data sets used in the research and related performance evaluation indicators. The fourth part is to test the designed model to verify the ability of the design algorithm.

## Related Works

In the course of consumer behavior, experiential education can help students to better understand consumer satisfaction with products and enhance the teaching experience. With the continuous development of AI, recognition technology has been applied in many fields. Fan (2021), from the perspective of criminal psychology, applied the convolutional neural network (CNN) in deep learning to predict actual violations and build a crime prediction model. The research results showed that the designed model has a prediction accuracy of 96% for the five types of crimes, which provides a reference for crime prevention research (Fan, 2021). Yang et al. (2021) studied emotion recognition systems based on commercial cloud services under human facial expressions and compared the accuracy of five common well-known commercial emotion recognition services. The research results showed that these commercial recognition systems have different processing results when processing distorted images and some suggestions are provided for developers in response to the existing problems (Yang et al., 2021). Taghikhah et al. (2021) studied the influence of consumer behavior on product purchase decisions. Supervised and unsupervised machine learning algorithms were used and behavioral theory was combined to quantify the intentions and behaviors of consumers, so as to realize the combination of emotional factors and normative cues to predict the buying behavior of consumers (Taghikhah et al., 2021). Lixăndroiu et al. (2021) studied the influence of personality characteristics and attitudes toward the Internet on traditional electronic online shopping and augmented reality E-commerce. The results showed that in the case of augmented reality, the purchase intentions of consumers for online shopping are significantly higher and personality traits have been proven to predict impulse purchases (Lixăndroiu et al., 2021).

However, there is a lot of research on deep learning in consumer behavior analysis. Hung and Chang (2021) studied a method of applying transfer learning to DNNs and applying it to computer vision and proved its effectiveness by performing facial emotion and named entity recognition tasks. This method was applied to 20 participants who observed familiar and unfamiliar markers, and the results also support the model, which can be used as an effective tool for exploring the familiar and unfamiliar marker data of participants. This method can be applied to the study of other perception processes in cognition and computer neuroscience (Hung and Chang, 2021). Kim et al. (2021) used deep hybrid learning algorithms to analyze customer-oriented data and predicted customer buyback behavior of smartphones of the same brand. The research results show that the model based on the deep hybrid learning algorithm has a prediction accuracy of more than 90%, which provides an effective reference for innovating future marketing strategies (Kim et al., 2021). Luo and Xu (2021) studied the impact of website reviews on the dining decisions of customers during coronavirus disease 2019 (COVID-19) period and combined the deep learning with customer reviews to evaluate the characteristics of the restaurant. Research results showed that the designed algorithm is superior to traditional machine learning algorithms in sentiment classification and comment rating prediction (Luo and Xu, 2021). Kottursamy (2021) studied the role of facial expression recognition in social science and HCI, discussed various common deep learning algorithms for emotion recognition, and established a new CNN model based on the eXnet library to achieve higher facial recognition accuracy.

In summary, from the current research status, AI technology and HCI technology have been widely used in sentiment analysis and certain results have also been achieved. To enhance the experience of students in consumer behavior courses and more accurately predict the psychological behaviors of consumers, it is proposed to collect consumer facial expression information through HCI technology and to use AI recognition technology for the context of E-commerce. The psychological behavior represented by the facial expressions of the consumer is judged to obtain the satisfaction of people with the product to enhance the teaching experience of student. In addition, DNN is used to analyze and predict the internal laws of consumer behavior, so as to provide technical support for the realization of precision marketing of products in the context of E-commerce.

## Materials and Methods

### Application of Human–Computer Interaction and Recognition Technology in the Course of Consumer Behavior Under the Background of E-Commerce

As an independent and systematically applied science, consumer behavior appeared after the capitalist industrial revolution, with the rapid development of the commodity economy, increasingly acute market problems, and intensified competition. From the end of the nineteenth century, theories about consumer behavior and psychology began to appear. At the beginning of the twentieth century, the increase in labor productivity after the industrial revolution caused production capacity to exceed market demand. The competition among enterprises on the market has also intensified. Therefore, some enterprises have begun to pay attention to the stimulation of consumer demand and the marketing strategy of their products. In addition, some scholars began to theoretically study the relationship between commodity demand and sales, and the relationship between consumer behavior, psychology, and sales strategies based on the needs of corporate sales. However, the emergence of E-commerce has also changed the traditional transaction mode, enabling merchants and consumers to communicate in real-time.

When websites or customers provide relevant technical support, visual communication can be realized, which brings great convenience to both consumers and businesses. Merchants can display products more comprehensively, and consumers can also have a deeper understanding of products and make purchases according to their needs. More importantly, the individual needs of consumers can also be met. Compared with the traditional transaction model, it is more efficient and can meet the time and energy needs of many customers who are busy with work or are unwilling to go to physical stores. In addition, the advantages of a wide variety of products, low prices, and greater convenience also meet the needs of people who do not go shopping. However, this kind of poorly targeted information browsing will undoubtedly consume more energy and time for consumers, and it is also difficult for consumers to truly choose their favorite products from more commodities. In addition, due to the development of technology, many companies use crawlers to obtain data or analyze shopping lists of consumers to find out what consumers want to buy, buying habits, and consumption preferences as much as possible. Companies can further refine product requirements, classify consumers, and conduct precision marketing and recommendation services.

But, this caused confusion for students in the course of consumer behavior to understand consumer satisfaction with products. Experience is procedural, personal, and non-teaching. It is a process full of personality and creativity. Through experiential education, a simulated environment can be created, allowing students to enter the scene and face some problems and obstacles in the created environment, and allow students to find solutions to problems, thereby enhancing the teaching experience in this process. Therefore, it is proposed to use AI technology to understand the consumption experience of consumer in time in the course. HCI realizes the dialog between humans and machines in an effective way through the input and output devices of the computer. However, HCI and recognition technology have been widely studied in many fields. Therefore, it aims to explore the psychological state of consumers, so as to help E-commerce platforms recommend products that can better meet consumer needs (Zeng et al., 2018; Georgescu et al., 2019). The facial expressions of consumers contain a wealth of information about human behavior. As a carrier of information, facial expressions can show the psychological state of people to a certain extent. In addition, automatic facial expression analysis (AFEA) can extract features of facial expression information, and classify and understand them according to the understanding and thinking styles of people. In addition, human prior knowledge of emotional information is used to make computers associate, think, and reason to further analyze human emotions and psychology (Wu et al., 2019; He et al., 2021).

Under normal circumstances, the facial expressions of consumers will change with their mental state. For example, when consumers find a product they like, they will show a happy expression, and when they do not like it, they will shake their heads or show a relatively calm expression. When facial expressions are recognized, details are the key to distinguish facial expressions, so it is necessary to be able to analyze the subtle deformation of facial expressions (Pereira et al., 2019; Yuan and Wu, 2020; Song et al., 2021). Therefore, the key feature points of the face image training set need to be calibrated, which can be expressed as follows:


(1) $$L=\left\{\left(I_{i},X_{i}|i=1,\ldots ,N;X_{i}={\left(x_{i0},y_{i0},\ldots ,x_{i\left(n-1\right)},y_{i\left(n-1\right)}\right)}^{T}\right)\right\}$$

where *N* represents the number of training samples, *n* stands for the number of pre-set key feature points, and *L* expresses that each shape vector *X*_*i*_ is formed by coordinates in a series of *n* key feature points manually demarcated on training image *I*_*i*_.

### Improvement of the Deep Neural Network Model

Deep neural network is the basis of deep learning, and it increases the number of network layers in the middle layer compared with a relatively shallow neural network. It can learn more useful feature information in the data for classification prediction. With the increase in number of hidden layers, the number of neurons will grow accordingly, so that the network model can learn more features from the data, thus facilitating the follow-up work. Moreover, DNN has been applied in many research fields, especially in the recognition, which can greatly reduce the error rate of speech recognition (Wu and Wu, 2017; Wenzel et al., 2019; Wu W. et al., 2020). The main parts of the DNN model include the input layer, the hidden layer, and the output layer. Furthermore, more hidden layers can enhance the expression ability of the model and increase its complexity. For the output layer, there is usually one output neuron, but there are multiple outputs in the DNN, so that the model can be flexibly applied to classification regression and other machine learning fields such as dimension-reduction and clustering (Barić et al., 2021; Gross et al., 2021).

**FIGURE 1:**
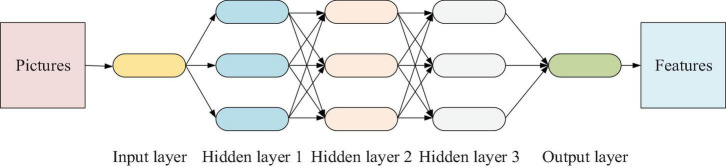
Basic structure of deep neural network (DNN).

Deep neural network replaces the traditional shallow neural network. This is because the shallow neural network is prone to fit during training and the training speed is slow. More importantly, DNN is a fully connected structure between upper and lower neurons. The shallow neural network improves the back-propagation training mechanism and uses layer-by-layer training to increase the training speed, avoiding network fitting (Segler et al., 2018; Liu et al., 2021). Figure 1 shows the basic structure of DNN.

The training speed of DNN is faster than that of the shallow neural network, but the DNN model is adopted to simulate multiple non-linear fittings from the underlying network in the current big data environment to unearth the hidden features in the data. Thus, there are still some shortcomings after it shows the feature learning ability and the performance of the network in specific issues has to be improved (Chen, 2019; Wu and Song, 2019). In particular, DNN contains many parameters, such as the number of hidden layers, the number of neurons, and the activation function, all of which should be continuously optimized. Moreover, as the increase on the number of hidden layers, the corresponding network weights will become larger and larger, which further leads to a longer training time. Therefore, the DNN model is improved with a view to the disadvantages of the model and the pursuit of high efficiency in practical application, so as to obtain the region-DNN (rDNN) model (Kanagaraj and Priya, 2021). The collected negative samples are ozonized randomly by the rDNN model, and then the proportion of unbalanced data is analyzed (Rogoza et al., 2018; Zhu et al., 2018).

**FIGURE 2:**
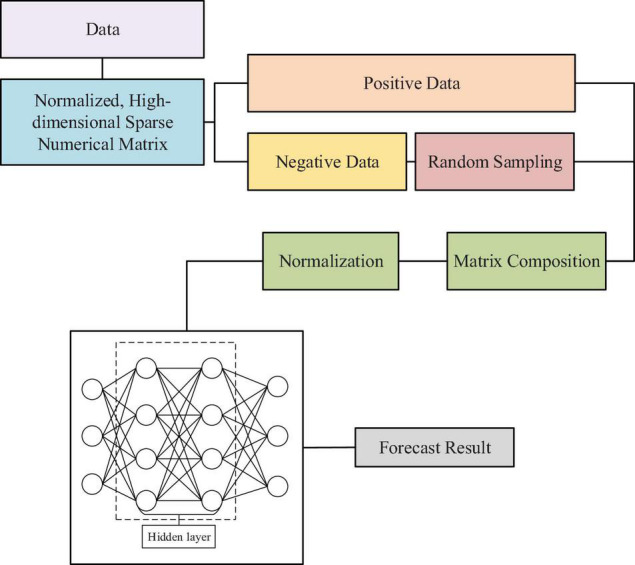
The region-deep neural network (rDNN) model (Kanagaraj and Priya, 2021).

Relevant studies reveal that the rDNN model can accelerate the training speed of the model, eliminate the redundancy of negative data samples, and automatically learn data features from the bottom layer of the neural network, thereby digging out more valuable information in the data. The improved rDNN model is shown in Figure 2.

The sample data should be processed based on the DNN model to improve the training ability of subsequent DNN models. Moreover, it is necessary to select an appropriate activation function when the rDNN model is used because of its large number of parameters, so as to retain data features to the maximum extent (Wu Y.J. et al., 2020). The frequently used activation functions include Sigmoid and ReLU functions and Sigmoid is well applied in the neural network model, and its expression is shown in the following equation:


(2) $$f\left(x\right)=\frac{1}{1+exp\left(-x\right)}$$

where *x* represents the input value and can take any function value, while the range of output value *f*(*x*) is in (0,1). For the ReLU function, it is a piecewise function. The output value is 0 when the input value is less than 0, and the output value is the input value when the input value is greater than 0. It can be expressed in Equation (3):


(3) g(x)=max(0,x)

Comparison of the two activation functions indicates that the saturated part of the Sigmoid function in the positive and negative gradient is close to zero (Bai et al., 2021; Jin et al., 2021). However, the gradient of the ReLU function greater than 0 is a constant and can prevent the gradient from dispersion (Zhou et al., 2020; Xiong et al., 2021). During the calculation, there is a faster calculation speed of the derivative of the ReLU function, so the ReLU function is more used in convolution kernels of CNN (Zuo et al., 2017; Lohse et al., 2020).

### Experimental Data Set

In order to test the accuracy of the recognition algorithm designed in this article, the Jaffee database^1^ is used as the training and testing database for facial expression recognition on Matlab R2018a. The designed algorithm is used to extract the image features, and then the classifier is used to classify the fusion results. The Jaffe database is used for facial expression recognition. It includes 213 Japanese female faces (each face has a definition). There are 10 people in the database, with 7 facial expressions for each person, including 6 basic expressions and 1 neutral expression (Angry, Disgust, Fear, Happy, Sad, Surprise, and Neutral).

**FIGURE 3:**
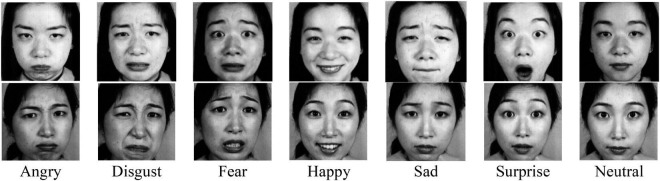
Examples from modified Jaffe database (Lyons et al., 2020). Reproduced with permission of Jaffe database.

To better analyze the facial features of consumers, the face image in the Jaffe database is preprocessed, and the face area of the image is cropped to remove unnecessary interference. Then, the obtained face images are re-saved in the revised Jaffe database for the subsequent experimental test process. Facial expressions of people use recognition technology to extract facial features to help students to understand the experience of consumers of products in the course of consumer behavior. In this study, the judgment algorithm is used to compare the emotional characteristics represented by different types of images to verify the judgment result of the designed algorithm on the mental state of consumers. Figure 3 shows some pictures in the revised Jaffe database.

### Model Parameters and Evaluation Plan

In the training process of the model, negative samples and positive samples are also cited. The ratio of negative samples and positive samples is uniformly defined as negative sample/positive sample, referred to as *N*/*P* ratio (Xue et al., 2020; Zhang et al., 2021). When the parameters of the rDNN model are set, the model structure is 128-64-64-30-2, the objective function is binary_crossentropy, and the maximum number of iterations is 500. After randomly sampling the negative data samples, the positive data samples are used to form the Random data set with a learning rate of 0.01.

The following evaluation indicators are adopted to evaluate and compare the classification effect of the classifier. True positive (TP) means that positive samples are classified as positive samples; false positive (FP) means that negative samples are classified as positive samples; true negative (TN) refers to that positive samples are classified as negative samples; and false negative (FN) means that negative samples are classified as negative samples.

(1) Accuracy describes the classification accuracy of the classifier.


(4) $$Accuracy=\frac{TP+FN}{TP+FP+TN+FN}$$

(2) Area under curve (AUC) is a two-category evaluation method commonly used in machine learning, and its direct meaning is the area under the receiver operating characteristic curve (ROC). The calculation equation is given as follows:


(5) $$AUC=\frac{\sum_{i\in positiveClass}rank_{i}-\frac{M\left(1+M\right)}{2}}{M\times N}$$

In the equation above, M represents the number of positive samples, N represents the number of negative samples, and *rank*_*i*_ represents the number of samples whose predicted probability exceeds.

## Results and Discussion

### Analysis of Facial Expression Recognition Results

**FIGURE 4:**
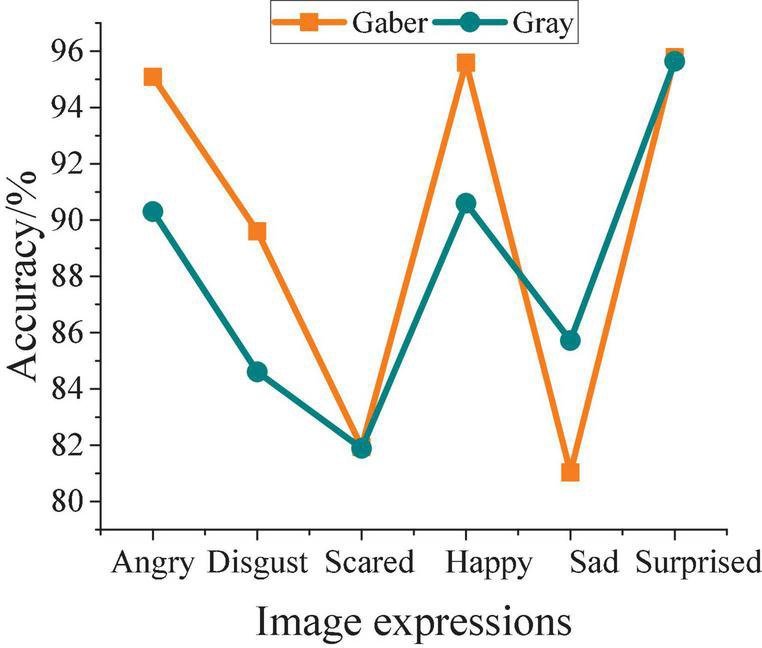
Results of facial expression recognition accuracy.

The recognition accuracy of Gabor features and gray-scale difference features under different expressions is compared and analyzed after the image expressions were recognized. The results are shown in Figure 4.

As shown in Figure 4, for anger, disappointment, happiness, and surprise, the expression of Gaber features will make the recognition accuracy higher; and for surprise, the results of the two are the same; and for sadness, the gray difference feature shows better recognition accuracy than the Gaber feature. However, the overall recognition accuracy of expressions under Gaber features is high from the overall research results. Therefore, the Gaber feature is selected as the feature analysis method of the research algorithm in the following experiments.

### Predictive Performance Analysis of Deep Learning Model

**FIGURE 5:**
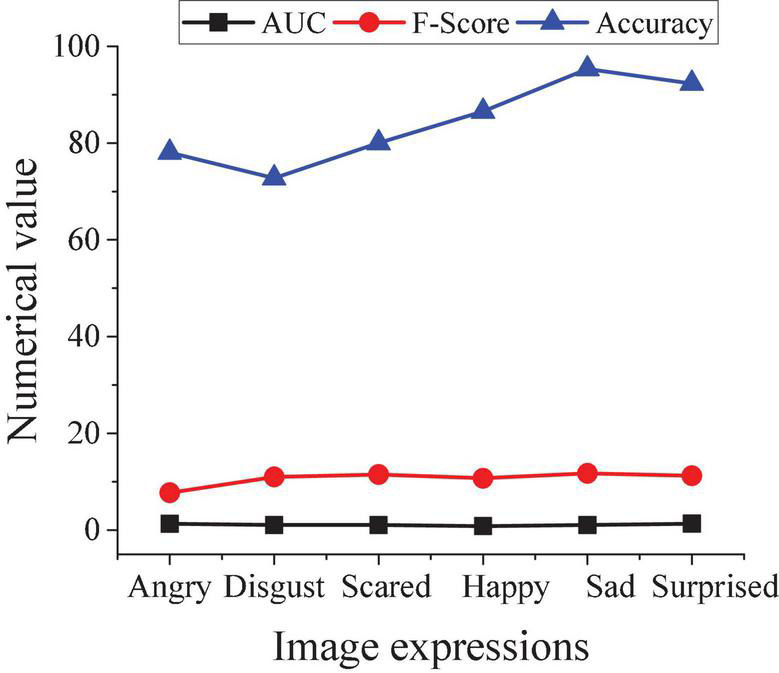
Comparison on the different deep learning models.

During the specific analysis of the model performance, the points on the ROC reflect the sensitivity to the stimulus of the same signal. The horizontal axis of the curve indicates the specificity of FP rate (FPR), while the vertical axis represents the sensitivity of TP rate (TPR). The area under the curve (AUC) refers to the area under the receiver operating characteristic (ROC). Therefore, the AUC value can be applied to evaluate the prediction of the model. In addition, the different deep learning models are compared and analyzed when the rDNN model is used for prediction. The specific results are given in Figure 5.

The above comparison reveals that the performance of the random forest model is better based on the AUC and *F* value, and it can integrate the results of the multiple decision trees. On the basis of the characteristics of the stochastic and non-linear of the data, it reflects better study effect and fast training speed, can deal with high-dimensional data, and express a good prediction effect when the data do not need to make big changes. However, the neural network can effectively simulate the non-linear characteristics of the data and has strong generalization ability, so it is easy to fall into the local minimum value. That is why, there is a big space for improvement of this method. These methods are superior to the logistic regression model; though it is classic and commonly used when dealing with classification, had no good effect in classifying the consumer behaviors. This is because the logistic regression model belongs to the linear regression model, and the stochastic and non-linear characteristics are stronger in the experimental data, thus influencing the implementation effect.

The *F*-value is extremely sensitive to sparse data, leading to the result value is not high. What’s more, Figure 5 also indicates that the accuracy of the three DNN models is higher than that of the other three models. Such results suggest that the recall rates in the logistic regression, neural network, and random forest model are low, which indicate that three classifiers are more sensitive to data and focus more on the learning of negative data in the model training, thereby neglecting the learning of positive data. Besides, more attention should be paid to the samples with less category data in the prediction. The AUC value of the rDNN model proposed is higher than that of the other models, while the *F* value is the weighted harmonic mean of accuracy and recall rate to synthetically evaluate experimental results and quality.

### Test to Compare the Model Performances

**FIGURE 6:**
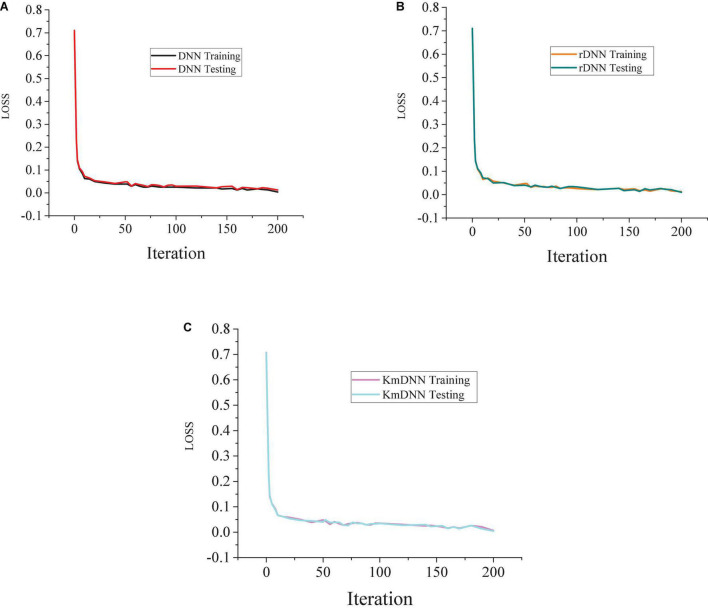
Analysis of model iteration results. **(A)** Testing results of DNN. **(B)** Testing results of rDNN. **(C)** Testing results of Kmean-DNN (KmDNN).

When the iterative process of DNN, rDNN, and Kmean-DNN (KmDNN) is further analyzed, it is found that the model tends to be stable when the iteration times reach 170 during the iteration process of the verification set in the three models. Figure 6 shows the analysis results of model iteration.

**FIGURE 7:**
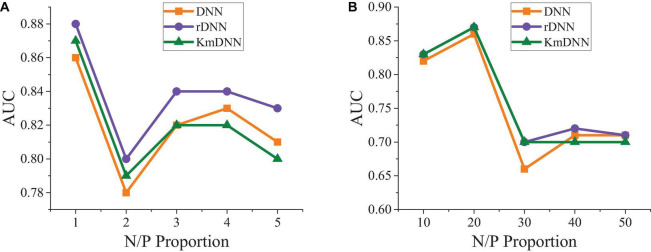
Comparison on prediction effects under different positive and negative sample proportions. **(A)** N/P = 1–5. **(B)** N/P = 10–50.

The above results show that the rDNN model is better than the KmDNN and DNN model. In the analysis and prediction of consumer behaviors, the rDNN model also retains the original DNN the ability to automatically learn the deep features of data and abstract high-level features, so it shows good practicability in reducing model training. When the proportion of positive and negative samples is analyzed, the results given in Figure 7 can be obtained.

Figure 7 reveals that changes in proportions of positive and negative samples show little influence on the prediction effect and the prediction result is optimal when the proportion is 3.

**FIGURE 8:**
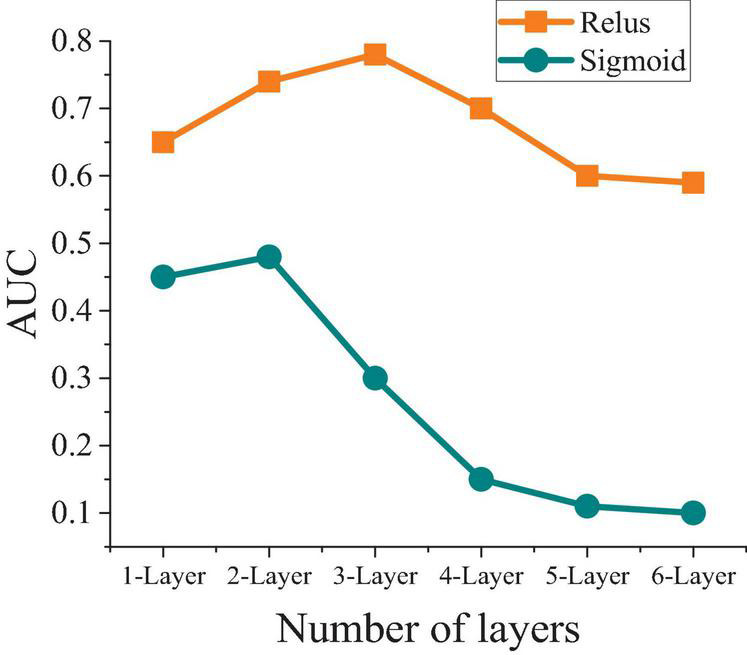
Comparative analysis of different activation functions under different hidden layers.

When different activation functions are analyzed, the results are obtained. Figure 8 shows the comparative analysis of different activation functions under different hidden layers.

Figure 8 indicates that the prediction effect of ReLU function is better than that of Sigmoid function, and the number of hidden layers shows a small influence on the model. When the number of layers is 2, the network model of the Sigmoid function has a good prediction effect, but the number of layers increases gradually and the prediction effect shows a downward trend. When the ReLU function is applied, the prediction effect is best if the number of layers is 3. As the number of layers increases, the prediction effect also decreases, but the overall range is small and the result is relatively stable.

### Case Analysis

Finally, the satisfaction of 10 consumers with the recommended products is analyzed to verify the actual performance of the design model according to consumer psychology and the results are shown in Table 1.

**TABLE 1:** Analysis of recommended product satisfaction.

Customer number	Gender	Detail satisfaction	Detail deficiency	Average satisfaction
1	Male	93.0%	Color	93.4%
2	Female	95.3%	Size	94.1%
3	Male	95.0%	Size	93.8%
4	Female	92.7%	Color	92.7%
5	Female	93.5%	Color	93.3%
6	Female	93.6%	Size	94.5%
7	Male	92.8%	Color	93.6%
8	Female	95.4%	Size	93.7%
9	Male	96.1%	Size	92.9%
10	Male	93.6%	Color	93.0%

Table 1 shows that the satisfaction of consumers with the recommended products is higher than 92.7%, which can prove the effectiveness of the proposed method. In summary, the facial expression recognition technology applied here can more accurately identify the facial expression information of consumers, and predict their consumption preferences through data analysis, thereby recommending products with higher satisfaction. When the prediction model is analyzed, it is found that the rDNN model shows a better prediction effect.

## Discussion

In the course of consumer behavior, students need to analyze the meaning of consumer behavior in combination with psychology-related theories, so as to better design products and formulate marketing strategies. In the context of the development of the mobile Internet, it should improve the personalized service capabilities of online shopping activities in e-commerce. In the research process, it is proposed to use AI technology to realize experiential education for students in the course of consumer behavior. In the application of consumer psychology, it is proposed to collect facial expression data of consumers in the shopping process through the form of HCI. Based on DNN technology, the collected facial expression information is recognized and analyzed, the psychological behavior of consumers in the shopping process is judged and then the satisfaction of consumers with the product is predicted.

To realize the recognition of consumer facial expression information, the DNN algorithm is applied to facial recognition by analyzing the current research status of related fields in the research, and a consumer facial expression recognition algorithm based on the rDNN algorithm is established. According to the identification results, the psychological behavior and product satisfaction of consumers during the shopping process are judged. Experiments have proved that the designed model has a good performance in dealing with facial expression recognition. Compared with the traditional forecasting model, the forecast accuracy of the designed model is increased by 10%. This suggests that the designed model shows better performance than similar models and can be better applied to the shopping demand prediction of consumers. The results can provide a reference for students to conduct experiential education to understand the user experience of consumers in the service process in the course of improving consumer behavior.

## Conclusion

To explore the understanding of the consumption psychology of consumers in the course of consumer behavior under E-commerce, it is proposed to use AI to recognize the facial expressions of consumers and to apply HCI to obtain consumer satisfaction with the product. Finally, the improved DNN is used to predict the psychological behaviors of consumers, so as to realize the formulation of precision marketing strategies in experiential courses. The experimental results also prove that the design algorithm shows a better predictive effect than similar algorithms. The research results can be applied to the analysis of consumer psychological behavior in E-commerce scenarios, so as to help students provide consumers with a personalized consumption experience. In addition, choosing appropriate predictive models and applying more advanced technologies can expand the scope of research, thereby offering companies and researchers with more research data, and providing ideas for constructing more effective research models.

However, there are some shortcomings in this work. Due to the small number of samples in the data set, the designed model has limited types of facial expression recognition. The current model can only recognize and judge 7 kinds of expressions. Moreover, there is still a lack of reliable analysis of consumer gesture and motion recognition in the current research, which plays an important role in the follow-up research of HCI. Therefore, in the subsequent consumer behavior research, the types of expressions in the data set will be further expanded, and the recognition and analysis of consumer gestures and shapes will be studied, so as to more accurately predict consumer psychological behavior.

## Data Availability Statement

The raw data supporting the conclusions of this article will be made available by the authors, without undue reservation.

## Ethics Statement

The studies involving human participants were reviewed and approved by the Peking University Ethics Committee. The patients/participants provided their written informed consent to participate in this study. Written informed consent was obtained from the individual(s) for the publication of any potentially identifiable images or data included in this article.

## Author Contributions

All authors listed have made a substantial, direct, and intellectual contribution to the work, and approved it for publication.

## Conflict of Interest

The authors declare that the research was conducted in the absence of any commercial or financial relationships that could be construed as a potential conflict of interest.

## Publisher’s Note

All claims expressed in this article are solely those of the authors and do not necessarily represent those of their affiliated organizations, or those of the publisher, the editors and the reviewers. Any product that may be evaluated in this article, or claim that may be made by its manufacturer, is not guaranteed or endorsed by the publisher.

## Footnotes

1
http://www.kasrl.org/jaffe.html

## References

[B1] Bai B. Guo Z. Zhou C. Zhang W. Zhang J. (2021). Application of adaptive reliability importance sampling-based extended domain PSO on single mode failure in reliability engineering. *Inform. Sci.* 546 42–59. 10.1016/j.ins.2020.07.069

[B2] Barić D. Fumić P. Horvatić D. Lipic T. (2021). Benchmarking attention-based interpretability of deep learning in multivariate time series predictions. *Entropy* 23:143. 10.3390/e23020143 33503822 PMC7912396

[B3] Campbell M. C. Winterich K. P. A. (2018). Framework for the consumer psychology of morality in the marketplace. *J. Consum. Psychol.* 28 167–179.

[B4] Chen M. (2019). The impact of expatriates’ cross-cultural adjustment on work stress and job involvement in the high-tech industry. *Front. Psychol.* 10:2228. 10.3389/fpsyg.2019.02228 31649581 PMC6794360

[B5] Cichy R. M. Kaiser D. (2019). Deep neural networks as scientific models. *Trends Cogn. Sci.* 23 305–317. 10.1016/j.tics.2019.01.009 30795896

[B6] Fan Y. (2021). Criminal psychology trend prediction based on deep learning algorithm and three-dimensional convolutional neural network. *J. Psychol Afr.* 31 292–297. 10.1080/14330237.2021.1927317

[B7] Feng S. Zhou H. Dong H. (2019). Using deep neural network with small dataset to predict material defects. *Mater. Des.* 162 300–310. 10.1016/j.matdes.2018.11.060

[B8] Georgescu M. I. Ionescu R. T. Popescu M. (2019). Local learning with deep and handcrafted features for facial expression recognition. *IEEE Access.* 7 64827–64836. 10.1109/access.2019.2917266

[B9] Gross N. Byers V. Geiger S. (2021). Digital health’s impact on integrated care, carer empowerment and patient-centeredness for persons living with dementia. *Health Policy Technol.* 10:100551. 10.1016/j.hlpt.2021.100551

[B10] He L. Chan J. C. W. Wang Z. (2021). Automatic depression recognition using CNN with attention mechanism from videos. *Neurocomputing* 422 165–175. 10.1016/j.neucom.2020.10.015

[B11] Hung J. C. Chang J. W. (2021). Multi-level transfer learning for improving the performance of deep neural networks: theory and practice from the tasks of facial emotion recognition and named entity recognition. *Appl. Soft Comput.* 109:107491. 10.1016/j.asoc.2021.107491

[B12] Jin Y. Qi W. Zong G. (2021). Finite-time synchronization of delayed semi-Markov neural networks with dynamic event-triggered scheme. *Int. J. Control Autom. Syst.* 19 2297–2308. 10.1007/s12555-020-0348-2

[B13] Kanagaraj K. Priya G. G. L. (2021). A new 3D convolutional neural network (3D-CNN) framework for multimedia event detection. *Signal Image Video Process.* 15 779–787. 10.1007/s11760-020-01796-z

[B14] Kell A. J. Yamins D. L. Shook E. N. Norman-Haignere S. V. McDermott J. H. (2018). A task-optimized neural network replicates human auditory behavior, predicts brain responses, and reveals a cortical processing hierarchy. *Neuron* 98 630–644. 10.1016/j.neuron.2018.03.044 29681533

[B15] Kim J. Ji H. G. Oh S. Hwang S. Park E. del Pobil A. P. (2021). A deep hybrid learning model for customer repurchase behavior. *J. Retail. Consum. Serv.* 59:102381. 10.1016/j.jretconser.2020.102381

[B16] Kim T. Y. Cho S. B. (2019). Predicting residential energy consumption using CNN-LSTM neural networks. *Energy* 182 72–81. 10.1016/j.energy.2019.05.230

[B17] Kottursamy K. (2021). A review on finding efficient approach to detect customer emotion analysis using deep learning analysis. *J. Trends Comput. Sci. Smart Technol.* 3 95–113. 10.36548/jtcsst.2021.2.003

[B18] Lamberton C. (2019). Toward a dignity architecture: the critical challenges of stigmatized-identity cues for consumer psychology. *J. Consum. Psychol.* 29 152–159. 10.1002/jcpy.1077

[B19] Liu Z. Lang L. Hu B. Shi L. Huang B. Zhao Y. (2021). Emission reduction decision of agricultural supply chain considering carbon tax and investment cooperation. *J. Clean. Prod.* 294:126305. 10.1016/j.jclepro.2021.126305

[B20] Lixăndroiu R. Cazan A. M. Maican C. I. (2021). An Analysis of the impact of personality traits towards augmented reality in online shopping. *Symmetry* 13:416. 10.3390/sym13030416

[B21] Lohse L. M. Robisch A. L. Töpperwien M. Maretzke S. Krenkel M. Hagemann J. (2020). A phase-retrieval toolbox for X-ray holography and tomography. *J. Synchrotron Radiat.* 27 852–859. 10.1107/S1600577520002398 32381790 PMC7206550

[B22] Luo Y. Xu X. (2021). Comparative study of deep learning models for analyzing online restaurant reviews in the era of the COVID-19 pandemic. *Int. J. Hosp. Manag.* 94:102849. 10.1016/j.ijhm.2020.102849 34785843 PMC8586815

[B44] Lyons M. J. Kamachi M. Gyoba J. (2020). Coding facial expressions with gabor wavelets (IVC Special Issue). *arXiv:2009.05938.* 10.5281/zenodo.4029679

[B23] Pereira T. D. Aldarondo D. E. Willmore L. Kislin M. Wang S. S. H. Murthy M. (2019). Fast animal pose estimation using deep neural networks. *Nat. Methods* 16 117–125. 10.1038/s41592-018-0234-5 30573820 PMC6899221

[B24] Qian J. Song B. Jin Z. Wang B. Chen H. (2018). Linking empowering leadership to task performance, taking charge, and voice: the mediating role of feedback-seeking. *Front. Psychol.* 9:2025. 10.3389/fpsyg.2018.02025 30410461 PMC6209672

[B25] Rogoza R. Żemojtel-Piotrowska M. Kwiatkowska M. M. Kwiatkowska K. (2018). The bright, the dark, and the blue face of narcissism: the Spectrum of narcissism in its relations to the metatraits of personality, self-esteem, and the nomological network of shyness, loneliness, and empathy. *Front. Psychol.* 9:343. 10.3389/fpsyg.2018.00343 29593627 PMC5861199

[B26] Segler M. H. Preuss M. Waller M. P. (2018). Planning chemical syntheses with deep neural networks and symbolic AI. *Nature* 555 604–610. 10.1038/nature25978 29595767

[B27] Song J. Wang L. Liu Z. Liu M. Zhang M. Wu Q. (2021). Locality preserving and label-aware constraint-based hybrid dictionary learning for image classification. *App. Sci.* 11:7701. 10.3390/app11167701

[B28] Taghikhah F. Voinov A. Shukla N. Filatova T. (2021). Shifts in consumer behavior towards organic products: theory-driven data analytics. *J. Retail. Consum. Serv.* 61:102516. 10.1016/j.jretconser.2021.102516

[B29] Wenzel J. Matter H. Schmidt F. (2019). Predictive multitask deep neural network models for ADME-Tox properties: learning from large data sets. *J. Chem. Inform. Model.* 59 1253–1268. 10.1021/acs.jcim.8b00785 30615828

[B30] Wu W. Wang H. Wu Y. J. (2020). Internal and external networks, and incubatees’ performance in dynamic environments: entrepreneurial learning’s mediating effect. *J. Technol. Transf.* 46 1707–1733. 10.1007/s10961-020-09790-w

[B31] Wu W. Wang H. Zheng C. Wu Y. J. (2019). Effect of narcissism, psychopathy, and machiavellianism on entrepreneurial intention: the mediating of entrepreneurial self-efficacy. *Front. Psychol.* 10:360. 10.3389/fpsyg.2019.00360 30846958 PMC6393355

[B32] Wu Y. J. Liu W. J. Yuan C. H. (2020). A mobile-based barrier-free service transportation platform for people with disabilities. *Comput. Hum. Behav.* 107:105776. 10.1016/j.chb.2018.11.005

[B33] Wu Y. Song D. (2019). Gratifications for social media use in entrepreneurship courses: learners’ perspective. *Front. Psychol.* 10:1270. 10.3389/fpsyg.2019.01270 31214081 PMC6555126

[B34] Wu Y. Wu T. (2017). A decade of entrepreneurship education in the asia pacific for future directions in theory and practice. manage. *Manag. Decis.* 55 1333–1350. 10.1108/md-05-2017-0518

[B35] Xiong Z. Xiao N. Xu F. Zhang X. Xu Q. Zhang K. (2021). An equivalent exchange based data forwarding incentive scheme for socially aware networks. *J. Signal Process. Syst.* 93 249–263. 10.1007/s11265-020-01610-6

[B36] Xue X. Zhang K. Tan K. C. Feng L. Wang J. Chen G. (2020). Affine Transformation-enhanced multifactorial optimization for heterogeneous problems. *IEEE Trans. Cybern.* 1 1–15. 10.1109/TCYB.2020.3036393 33320820

[B37] Yang K. Wang C. Sarsenbayeva Z. Tag B. Dingler T. Wadley G. (2021). Benchmarking commercial emotion detection systems using realistic distortions of facial image datasets. *Vis. Comput.* 37 1447–1466. 10.1007/s00371-020-01881-x

[B38] Yuan C. H. Wu Y. J. (2020). Mobile instant messaging or face-to-face? Group interactions in cooperative simulations. *Comput. Hum. Behav.* 113:106508. 10.1016/j.chb.2020.106508

[B39] Zeng N. Zhang H. Song B. Liu W. Li Y. Dobaie A. M. (2018). Facial expression recognition via learning deep sparse autoencoders. *Neurocomputing* 273 643–649. 10.1016/j.neucom.2017.08.043

[B40] Zhang K. Zhang J. Ma X. Yao C. Zhang L. Yang Y. (2021). History matching of naturally fractured reservoirs using a deep sparse autoencoder. *SPE J.* 1 1–22.

[B41] Zhou Y. Tian L. Zhu C. Jin X. Sun Y. (2020). Video coding optimization for virtual reality 360-degree source. *IEEE J. Selected Top. Signal Process.* 14 118–129. 10.3390/s19030697 30744050 PMC6386839

[B42] Zhu M. Wang X. Wang Y. (2018). Human-like autonomous car-following model with deep reinforcement learning. *Transp. Res. Part C Emerg. Technol.* 97 348–368. 10.1016/j.trc.2018.10.024

[B43] Zuo C. Sun J. Li J. Zhang J. Asundi A. Chen Q. (2017). High-resolution transport-of-intensity quantitative phase microscopy with annular illumination. *Sci. Rep.* 7:7654. 10.1038/s41598-017-06837-1 28794472 PMC5550517

